# Neurodevelopmental Outcomes in Infants with Retinopathy of Prematurity and Bevacizumab Treatment

**DOI:** 10.1371/journal.pone.0148019

**Published:** 2016-01-27

**Authors:** Reyin Lien, Mu-Hsien Yu, Kuang-Hung Hsu, Pei-Ju Liao, Yen-Po Chen, Chi-Chun Lai, Wei-Chi Wu

**Affiliations:** 1 Division of Neonatology, Department of Pediatrics, Chang Gung Memorial Hospital, Taoyuan, Taiwan; 2 Chang Gung University, College of Medicine, Taoyuan, Taiwan; 3 Laboratory for Epidemiology, Department of Health Care Management, Chang Gung University, Taoyuan, Taiwan; 4 Department of Health Care Administration, Oriental Institute of Technology, New Taipei City, Taiwan; 5 Department of Business Administration, National Taiwan University, Taipei, Taiwan; 6 Department of Ophthalmology, Chang Gung Memorial Hospital, Taoyuan, Taiwan; University of Utah (Salt Lake City), UNITED STATES

## Abstract

**Purpose:**

The current study aims to investigate the neurodevelopment of premature infants after intravitreal injections of bevacizumab (IVB) for the treatment of retinopathy of prematurity (ROP) up to the age of 2 years.

**Methods:**

The study design was retrospective observational case series conducted at an institutional referral center. Infants with type 1 ROP were classified into 3 groups: laser only, IVB only, and a combination of IVB and laser treatment. Main Outcome Measures were neurodevelopmental outcomes of the patients after treatment were assessed by Bayley Scales for Infant Development.

**Results:**

Sixty-one patients who finished the neurodevelopmental survey were included. No detrimental effects on neurodevelopment were found in IVB group compared with the patients who received laser treatment only. The patients in the IVB + laser group had a higher incidence of significant mental (p = 0.028) and psychomotor (p = 0.002) impairment at 24 months than the patients in the laser group. The odds ratio of having severe psychomotor defects in the IVB + laser group was 5.3 compared with the laser group (p = 0.041). The causal source for the differences that were detected remained unknown due to lack of randomization in the study and accompanying bias in patient selection.

**Conclusions:**

Two years after laser and/or intravitreal injections of bevacizumab for infants with retinopathy of prematurity, no difference on neurodevelopment for those who received only bevacizumab versus only laser treatment were found. Those infants who required rescue therapy with laser or bevacizumab injection after initial, unsuccessful treatment showed some detrimental, neurodevelopmental effects.

## Introduction

Retinopathy of prematurity (ROP) is one of the primary causes of childhood blindness. In the later stages of ROP, neovascularization arises due to retinal immaturity, and this neovascularization leads to retinal traction and retinal detachment, which eventually affects vision. Neovascularization is mainly driven by vascular endothelial growth factor (VEGF) [1]. Currently, the recommended treatment for type 1 ROP as defined by the Early Treatment for Retinopathy of Prematurity Study is peripheral ablation by laser treatment [2]. Although laser treatment effectively halts the progression of stage 3 ROP to stage 4 ROP in 90% of patients [2], this treatment actually destroys large portions of the retina. A new treatment modality that is less traumatic to the retina is therefore urgently needed.

Bevacizumab (Avastin; Genetech Inc., San Francisco, CA) is a humanized anti-VEGF monoclonal antibody [3]. It is the first antiangiogenic agent for the treatment of metastatic colorectal cancer, and it has been used with good results in treating many retinopathies with VEGF up-regulation, including age-related macular degeneration [4,5], diabetic retinopathy [6–8], vitreous hemorrhage [9,10], neovascular glaucoma [11], and retinal vascular occlusion [12–14]. The VEGF level in the vitreous fluid has been shown to be highly elevated in ROP patients [15,16], suggesting the potential of intravitreal injections of bevacizumab (IVB) for the treatment of ROP.

IVB has been shown to be very effective in treating ROP. However, the BEAT-ROP study is the only prospective randomized study to use bevacizumab for ROP [17]. The BEAT-ROP study showed a significant benefit of IVB for zone I but not zone II disease in infants with stage 3+ retinopathy of prematurity compared with conventional laser therapy; however, it did not address the issue of safety because a large patient population of 2800 infants, a number difficult to achieve clinically, would be needed to address this issue. It also did not address the issue of long-term recurrence. Therefore, there are currently no definite conclusions for how best to use bevacizumab for ROP patients [18].

VEGF has been shown to play an important role in the neurodevelopment of newborn infants [19,20], and the impact of possible systemic VEGF suppression after IVB on the neurodevelopment of these patients is unknown. This study aimed to investigate the neurodevelopment of premature infants with severe ROP up to the age of 2 years after IVB. The outcomes were compared among the patients after laser treatment alone, IVB alone, and the combination of IVB and laser treatment.

## Materials and Methods

### Study Design

This was a retrospective study to assess neurodevelopment in extremely low birth weight (ELBW) infants after IVB or laser treatment for ROP. ELBW was defined as newborns with a birth weight of less than 1000 grams. The data were collected from Chang Gung Memorial Hospital in Taoyuan, Taiwan, and the study was approved by the Institutional Review Board of the Chang Gung Memorial Hospital, Taoyuan, Taiwan. If the patient had type 1 ROP as defined by the Early Treatment for Retinopathy of Prematurity Study [2], their parents or legal guardians were offered the choice of laser or IVB treatment after the benefits and risks of each had been thoroughly explained. The status of the off-label use of IVB for ROP treatment was also explained in detail. All parents consented to the treatments for their children with written document before the medical treatment provided to their children. During follow-up of their children, they signed additional consent for the participation of the study.

### Patient Enrollment

Patients with type 1 ROP who were treated with laser or IVB treatment from December 2007 to December 2010 were followed and collected for data analysis. The indications for treatment were type 1 ROP as defined by the Early Treatment for Retinopathy of Prematurity Study: zone I, any stage ROP with plus disease; zone I, stage 3 ROP without plus disease; or zone II, stage 2 or 3 ROP with plus disease [2]. If there was a recurrence of ROP or a lack of treatment response following IVB, the patients were offered additional laser treatment to stop the progression of ROP. If there was a recurrence of ROP or a lack of treatment response after laser treatment, the patients were offered additional IVB to stop the progression of ROP. The patients who received IVB as the first line treatment and had a favorable response to IVB with regression of ROP were classified as the IVB group. The patients who received laser treatment as the first line treatment and had a favorable response to laser treatment with regression of ROP were classified as the laser group, and those who received combined IVB and laser treatment with final regression of ROP were classified as the IVB + laser group.

The inclusion criteria for the current study were patients: with type 1 ROP who had received either laser, IVB, or combined treatment, with regression of ROP following treatment; who had been followed up with neurodevelopmental assessments for 2 years; and whose parents had signed consent forms. Patients with major congenital anomalies, and those who lost to follow-up in the 2-year study period, were excluded from the study. Relevant clinical events including Apgar score, hemodynamically significant patent ductus arteriosus that required medical or surgical intervention, necrotizing enterocolitis, bronchopulmonary dysplasia, sepsis, intraventricular hemorrhage (IVH), periventricular leukomalacia (PVL), the rate of inborn, use of antenatal steroids, number of days of ventilator used, respiratory dystress syndrome (RDS), and the postmenstrual age (PMA) of the first treatment were recorded.

### Treatment

The technique used for IVB was as previously described [21,22]. Briefly, the anesthesia involved an intravenous injection of midazolam (Dormicum, Cenexi SAS, Fontenay-sous-Bois, France) or fentanyl (Fentanyl-Fresenius, Bodene Limited, Port Elizabeth, South Africa) to sedate the infant before the IVB. Vital signs were monitored throughout the entire procedure. If the respiratory function of the infant was unstable, endotracheal intubation was performed to secure the airway. After the eyes had been prepared in a standard fashion using 5% povidone/iodine and topical antibiotics, 0.625 mg (0.025 ml) of bevacizumab was injected intravitreally via the pars plicata under intravenous sedation. The injection was performed initially with a 30-gauge needle directed perpendicularly to the globe, and then directed slightly toward the center of the eyeball after the needle passed the lens equator to prevent damaging the lens or retina. After the injection, retinal artery perfusion were checked, and the patients received topical antibiotics for 7 days.

The technique of laser treatment for ROP was as follows. Under intravenous sedation or general anesthesia, a diode laser with an indirect ophthalmoscopic delivery system (OcuLight^®^ SLx; Iridex, Mountain View, CA) was used to deliver laser spots with a near confluent pattern, and the end point for laser burns was a grade II gray burn applied to the entire avascular retina from the ridge of extraretinal proliferation to the ora serrata [23].

After the treatments, the patients were followed every 1 to 2 weeks until full vascularization of the retina was noted. Full vascularization was defined as vascularization as far as it would develop without an active fibrovascular component or clinically significant tractional elements [22].

### Neurodevelopmental Assessment

Neurodevelopmental assessments were part of standard care and the data were obtained from medical records. Neurodevelopmental outcomes in the ROP patients after IVB or laser treatment were assessed by Bayley Scales for Infant Development (II) [24]. A certified child psychologist who had experienced with this assessment and was blinded to the patient’s prior treatment performed the evaluations for the patients. The assessments were performed in the patients at the corrected age of 6, 12, 18, and 24 months. Each period had relevant items on the assessments. The assessments consisted of 2 parts: mental development index (MDI) and psychomotor development index (PDI), and each item was scored by the specialist. Briefly, the MDI estimated cognitive capacity (e.g., receptive and productive language skills, problem solving, memory, and imitation), and the PDI estimated gross and fine motor skills. The mean score of the Bayley Scales was 100, and a score of less than 70 (2 standard deviations below the mean) indicated a significant developmental delay [25].

### Statistics

Continuous variables were presented as the mean and standard deviation. The chi-square test was applied to examine associations between categorical variables in the study groups. The Shapiro-Wilk test was used to test the normality of the continuous variables in this study. An analysis of variance (ANOVA) was used to compare differences in numerical variables among the study groups, and the Kruskal-Wallis test was used for continuous variables with skew distribution. Scheffe’s post hoc test was used for post-hoc pairwise comparisons to identify the groups that were significantly different following ANOVA. Univariate analysis was performed to prescreen which time point of neuro-assessment appeared to have the maximal effect on poor neurodevelopmental outcomes among the study groups. Multiple logistic regression analysis was then performed for poor neurodevelopmental outcomes (MDI or PDI <70) among the study groups. The results were expressed as odds ratios (OR) after adjusting for sex, gestational age, and birth weight. When comparing cross-sectional neurodevelopmental outcomes among study groups, the missing data were excluded in the analyses.

Statistical Analysis System software version 9.2 (SAS Institute Inc.Cary, NC) was used for all data analyses. A p value less than 0.05 was considered to indicate statistical significance in this study.

## Results

**Fig 1:**
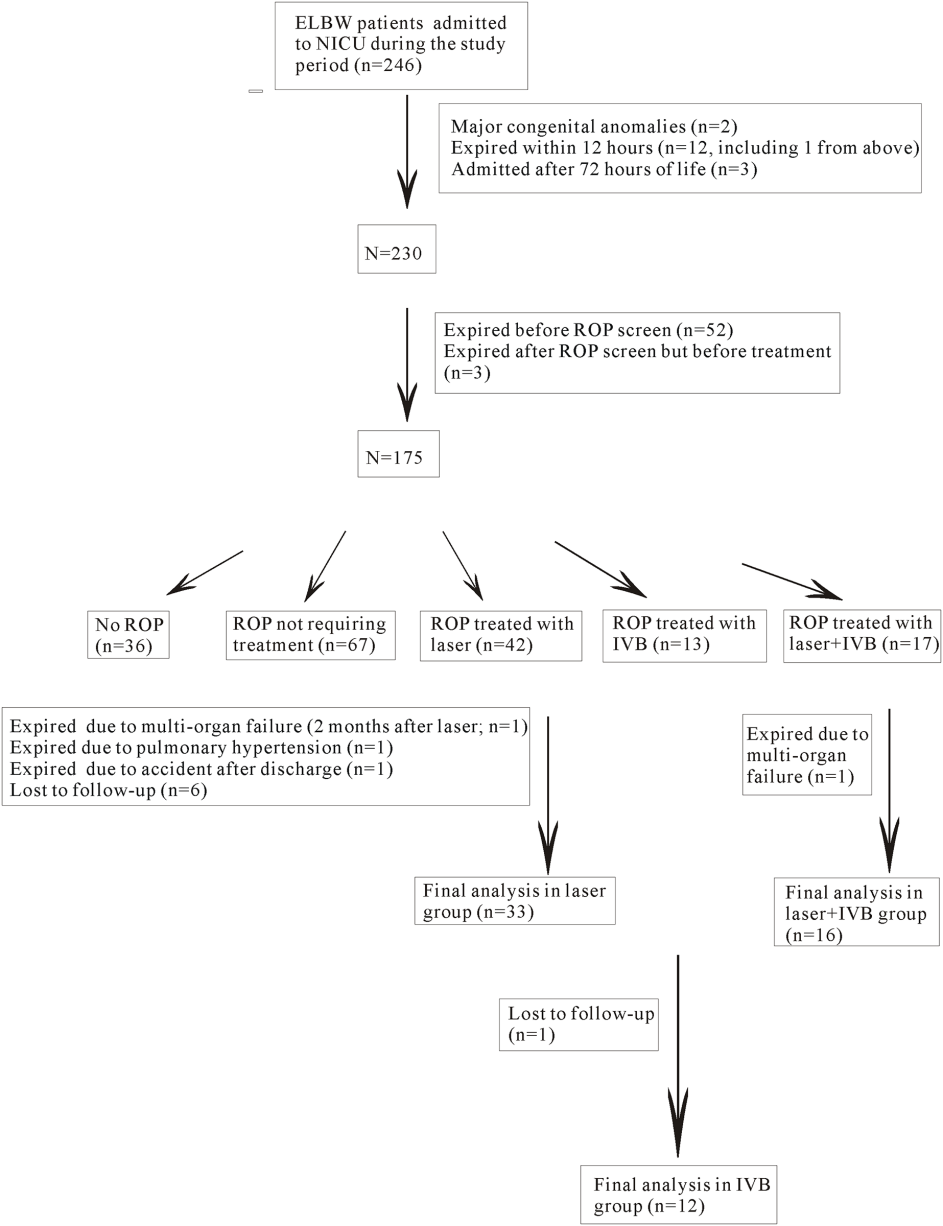
Flow chart showing the inclusion and exclusion of the patients during the study period. In total, 61 patients were enrolled and entered into the final analysis of the study.

A flow chart showing how the patients were enrolled and excluded from the study is presented in Fig 1. Sixty-one premature, ELBW patients who completed the neurodevelopmental assessments up to 2 years of age were included and entered into the final analysis. Among these patients, 33 received laser treatment only (laser group), 12 received IVB only (IVB group), and 16 received a combination of laser and IVB treatment (IVB + laser group). In the IVB + laser group, 9 patients (56%) received IVB first, but because of a lack of positive response or recurrence of ROP laser treatment was used as a salvage treatment. In the same group, 7 infants (44%) received laser treatment first, and then IVB was used as a salvage treatment due to a lack of treatment response or recurrent ROP. All of the patients except one in the laser group had bilateral ROP and therefore bilateral treatment. The mean number and range of surgical procedures were 1.4 ± 0.6 (range: 1 to 3), 1.1 ± 0.3 (range: 1 to 2), 2.6 ± 1.0 (range: 1 to 5) for laser group, IVB group, and IVB + laser group, respectively. All of the patients except 3 had regression of ROP after treatment. Two patients in the IVB + laser group and 1 patient in the laser group had progression to stage 4A ROP. After vitrectomy, the retinae of these 3 patients were attached. All of the patients in all of the groups had regression of ROP and an attached retina by the time of assessment. There were no differences in the gestational age at birth (p = 0.098), birth weight (p = 0.168), sex (p = 0.112), Apgar score at 1 minute after birth (p = 0.677), Apgar score at 5 minutes after birth (p = 0.778), the rate of hemodynamically significant patent ductus arteriosus (p = 0.772), the rate of necrotizing enterocolitis (p = 0.411), the rate of sepsis (p = 0.945), the rate of grade 1 or 2 IVH (p = 0.521), the rate of grade 3 or 4 IVH (p = 0.633), PVL (p = 0.239), the rate of inborn (0.801), use of antenatal steroids (0.484), number of days of ventilator used (p = 0.569) among the 3 study groups. The presence of zone 1 ROP was significantly higher in the IVB + laser group (p = 0.034). The rates of having zone 1 ROP were 15.2%, 25%, and 50% for the laser, IVB, and the IVB + laser groups, respectively. Bronchopulmonary dysplasia was present in all of the patients according to the definition of a previous paper,[26] and therefore the data were not entered into the statistical analysis. The demographics of the patients as well as the associated systemic risk factors are listed in Table 1.

**Table 1:** Demographics and the Systemic Risk Factors of the Patients Undergoing IVB, Laser, or Combined IVB and Laser Treatment.

	Laser (n = 33)	IVB (n = 12)	IVB+Laser (n = 16)	p value
Gestational age, mean (SD), weeks	25.6 (1.2)	25.0 (1.2)	24.8 (1.7)	0.098 ^a^
Birth weight, mean (SD), g	771.2 (91.2)	749.6 (155.1)	708.8 (92.5)	0.168 ^a^
Sex (M/F, n (%))	15 (45.5)/18 (54.5)	8 (66.7)/4 (33.3)	12 (75)/4 (25)	0.112 ^b^
Apgar score, 1 min, median (range)	4 (1, 8)	4 (1, 7)	4 (1, 7)	0.836 ^a^
Apgar score, 5 min, median (range)	6 (3, 9)	6 (1, 9)	6 (3, 9)	0.869 ^a^
Patent ductus arteriosus, n (%)	13 (39.4)	6 (50)	6 (37.5)	0.772 ^b^
Necrotizing enterocolitis, n (%)	2 (6.1)	0 (0)	2 (12.5)	0.411 ^b^
Sepsis, n (%)	15 (45.5)	6 (50)	7 (43.8)	0.945 ^b^
IVH, grade 1 or 2, n (%)	4 (12.1)	2 (16.7)	4 (25)	0.521 ^b^
IVH, grade 3 or 4, n (%)	5 (15.2)	2 (16.7)	1 (6.3)	0.633 ^b^
PVL, n (%)	0 (0)	0 (0)	1 (6.3)	0.239 ^b^
Presence of zone 1 ROP, n (%)	5 (15.2)	3 (25)	8 (50)	0.034 ^b^
Inborn, n (%)	29 (87.9)	10 (83.3)	13 (81.3)	0.811 ^b^
Use of antenatal steroids, n (%)	23 (69.7)	10 (83.3)	10 (62.5)	0.484 ^b^
Number of ventilator used (days)	88.9	88.5	95.2	0.569 ^a^
RDS, n (%)	33 (100)	12 (100)	16 (100)	1.00 ^b^
PMA of first treatment, mean (SD), weeks	35.3 (2.3)	35.2 (2.9)	33.7 (1.7)	0.063 ^a^

*Abbreviations*: *IVB* intravitreal injection of bevacizumab, *IVH* intraventricular hemorrhage, *PMA* post menstrual age, *PVL* periventricular leukomalacia, *RDS* respiratory distress syndrome, *ROP* retinopathy of prematurity, *SD* standard deviation

^a^ p values were calculated by Kruskal-Wallis test

^b^ p values were calculated by the chi-square test

In comparisons of the mean MDI scores, no significant differences were noted among the 3 treatment groups at 6 (p = 0.380), 12 (p = 0.793), and 18 (p = 0.100) months, however, significant differences were noted among the 3 groups at 24 (p = 0.028) months. In comparisons of the mean PDI scores, significant differences were noted among the 3 groups at 24 (p = 0.002) months. The scores were significantly different between the laser and the IVB + laser treatment groups based on the results of Scheffe’s post hoc test after multiple comparisons (Table 2).

**Table 2:** MDI and PDI Performed on Patients with Different Treatments up to 2 Years of Follow-up.

	Time	N	Laser	IVB	IVB+Laser	p value^a^
MDI, mean (SD)	6M	N = 58	95.0 (9.8)	93.9 (6.1)	90.3 (14.8)	0.380
	12M	N = 58	88.6 (11.2)	87.5 (9.0)	86.5 (7.0)	0.793
	18M	N = 55	90.0 (14.4)	81.0 (12.9)	81.3 (16.7)	0.100
	24M	N = 47	95.7 (14.5)	81.9 (18.6)	82.3 (19.2)	0.028 ^b^
PDI, mean (SD)	6M	N = 58	84.7 (14.5)	82.8 (13.3)	82.3 (12.3)	0.831
	12M	N = 57	78.5 (10.7)	70.4 (9.5)	72.0 (12.7)	0.056
	18M	N = 54	88.3 (16.5)	85.0 (14.8)	84.0 (19.3)	0.697
	24M	N = 47	98.0 (9.3)	88.1 (19.7)	79.6 (19.3)	0.002 ^b^

*Abbreviations*: *IVB* intravitreal injection of bevacizumab, *M* months, *MDI* mental developmental index, *PDI* psychomotor developmental index, *SD* standard deviation

^a^ p values were calculated by ANOVA

^b^ Scores were significantly different (p<0.05) between laser treatment and laser+IVB treatment based on the results of Scheffe’s post hoc test.

**Fig 2:**
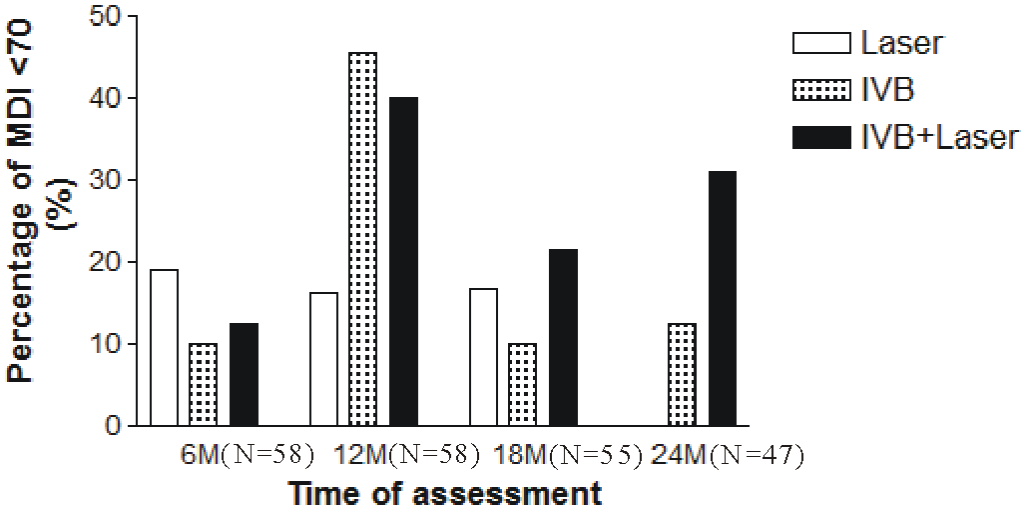
Comparison of significant mental developmental impairment in patients 2 years after various treatments for ROP. There were no statistically significant differences in significant developmental delays in MDI (MDI <70) among the 3 treatment groups at 6 (p = 0.066), 12 (p = 0.450), 18 (p = 0.226), or 24 (p = 0.057) months.

**Fig 3:**
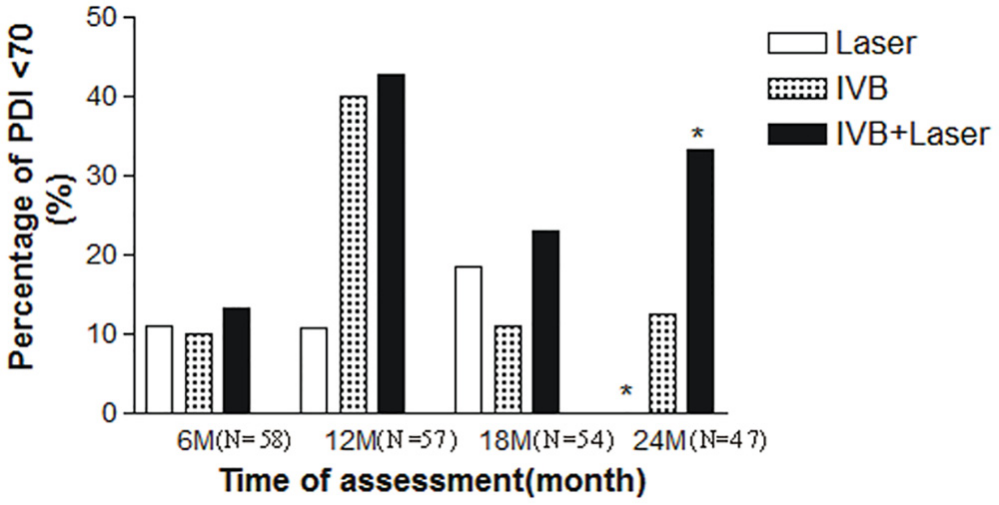
Comparison of significant psychomotor developmental defects in patients 2 years after treatments for ROP. Multiple-comparison analysis showed that the patients in the IVB + laser group had a higher incidence of severe psychomotor defects (PDI <70) at 24 (p = 0.010) months than the patients in the laser group (*indicates a significant difference).

Patients with Bayley Scales scores of less than 70 were considered to have significant developmental delays. There were no statistically significant differences in distributions of severe developmental delays in the MDI (MDI <70) among the 3treatment groups at 6 (p = 0.066), 12 (p = 0.450), 18 (p = 0.226), or 24 (p = 0.057) months (Fig 2). There were also no statistically significant differences in distributions of severe developmental delays in PDI (PDI <70) among the 3 treatment groups at 6 (p = 0.741), 12 (p = 0.087), and 18 (p = 0.760) months. However, the multiple-comparison analysis showed that the patients in the IVB + laser group had a higher risk for the occurrence of severe psychomotor impairment at 24 months compared to the patients in the laser group (p = 0.042) (Fig 3).

Univariate analysis was performed to prescreen which time regimen appeared to have the maximal effect on poor neurodevelopmental outcomes between the study groups. A maximal effect was found at 24 months on the MDI and 12 months on the PDI, and the data from those periods were then entered into multiple logistic regression analysis. The multiple logistic regression analysis of poor neurodevelopmental outcomes showed that the odds of having MDI <70 were 12.4 for the IVB group and 10.6 for the IVB + laser group compared with the laser treatment group. However, the analysis did not reach statistical significance (p = 0.084 and 0.075, respectively). The odds of having severe psychomotor developmental defects (PDI<70) were 4.7 for the IVB group and 5.3 for the IVB + laser group compared with the laser treatment group. The analysis did not reach statistical significance comparing the IVB group with the laser group (p = 0.075), however the analysis did reach statistical significance when comparing the laser + IVB group with the laser group (p = 0.041) (Table 3).

**Table 3:** Multiple Logistic Regression Analysis on Poor Neurodevelopmental Outcomes (MDI or PDI <70).

	MDI<70			PDI<70		
Treatment	OR ^a^	(95% CI)	p ^b^	OR ^a^	(95% CI)	p value ^b^
Laser	1	-	-	1	-	-
IVB	12.4	(0.7, 214.8)	0.084	4.7	(0.9, 25.9)	0.075
IVB + Laser	10.6	(0.8, 142.3)	0.075	5.3	(1.1, 26.3)	0.041

*Abbreviations*:, *CI* confidence interval, *IVB* intravitreal injection of bevacizumab, *MDI* mental developmental index, *OR* odds ratio, *PDI* psychomotor developmental index

^a^ Odds ratio was adjusted for sex, gestational age, and birth weight of the patients.

^b^ A maximal likelihood strategy, by which the data at the 24th month of MDI and the 12th month of PDI of the patients were selected and used to calculate the odds ratio of MDI or PDI <70.

We have compared the neurodevelopmental outcome between those infants treated with laser initially and then bevacizumab treatment compared to infants treated with bevacizumab treatment first and then laser. We did not find statistically significant difference between these 2 subgroups (data not shown). Also we did not find statistically significant difference in the ratio of severe neurodevelopmental defect between patients who had only one laser treatment and who had 2 laser treatments (data not shown).

## Discussion

Our results show that the patients in the IVB + laser group had a higher incidence of severe psychomotor developmental defects than the patients in the laser treatment alone group. The direct comparisons of MDI or PDI in the patients treated with laser treatment alone and IVB alone did not show significant differences in mental or psychomotor development up to 2 years of follow-up. Though there were no statistically significant differences found in the neurodevelopmental scores in the IVB group compared to laser group, there were certainly increased OR of abnormal MDI and PDI (at max effect) in the IVB group compared to the laser group (p values of 0.084 and 0.075, respectively). It is therefore a possible safety concern of using IVB on ROP patients and it is not known whether these effects will become more or less evident as we keep following up these patients. To the best of our knowledge, this paper is the first to evaluate the systemic development of children in children of Chinese decent after IVB with 2 years of longitudinal follow-up.

The cause of the worst neurodevelopmental outcome in the combined IVB and laser group remains to be investigated. The poorer outcome may be related to more surgical procedures being performed than in the other 2 groups of patients (1.4 ± 0.6, 1.1 ± 0.3, 2.6 ± 1.0 for laser group, IVB group, and IVB + laser group, respectively), and therefore a higher rate of sedation and anesthesia. In addition, a higher incidence of zone 1 ROP was noted in this group (15.2%, 25%, and 50% for the laser, IVB, and IVB + laser groups, respectively). Zone 1 ROP is usually associated with much worse outcomes compared with zone 2 ROP [2,17], and it is likely that patients with zone 1 ROP have a less mature organ system. A more immature avascular retina in zone 1 ROP may cause more anti-VEGF leakage into the systemic circulation. In addition, the destruction of the blood retina barrier by laser photocoagulation may result in a higher level of anti-VEGF leakage in the systemic circulation. Finally, the earlier the PMA at which the infants received treatment and retreatment was also a possible risk factor that could affect developmental outcome of these infants (Table 1). Infants in the IVB + laser group received the first treatment at approximately 1.5 weeks earlier that in the other treatment groups. The earlier treatment in the patients could be interpreted as these patients having worse eye diseases.

Recent studies in both animals [27,28] and humans [29,30] have found evidence of systemic bevacizumab exposure after IVB. In an animal study, IVB at an early age could result in more systemic bevacizumab exposure [27]. Sato and associates [29] found that systemic VEGF levels in premature infants were depressed for at least 2 weeks after the administration of either 1 or 0.5 mg IVB in ROP patients. Our recent study [30] has further shown that VEGF levels in ROP infants were depressed for 8 weeks after IVB. VEGF plays an important role in neurogenesis in embryos and preterm newborns. In previous reports, blocking VEGF-A expression has been shown to impair brain vascularization [20] and lead to neuron apoptosis in the retina [31]. In addition, VEGF has been found to be lower in preterm pups compared to term pups, and this has been proposed to relate to the neurodevelopmental delay and reduced growth of the cerebral cortex in premature infants [19]. Since neurogenesis may continue in the third trimester [19], further inhibition of serum VEGF in preterm newborns may have long-term effects on the development of the central nervous system and other systems. However, more research is needed to verify these speculations because anti-VEGF treatments are usually used for once or short term in these ROP patients.

The assessment of neurodevelopment after IVB for ROP has been conducted by few studies. Martínez-Castellanos et al [32] did not show any neurodevelopmental impact 5 years after the use of IVB. Banker et al (Banker AS; Banker DA. Anatomical, functional, OCT and neurodevelomental analysis outcomes of Intravitreal Bevacizumab injection without laser for retinopathy of prematurity: 6 year follow-up. APAO, Hyderabad, India 2013) also did not find any signs of developmental delay in infants 6 years following the use of IVB. However, these studies and ours were limited by a small number of patients and a non-randomized study design. Other anti-VEGF agents with a shorter systemic half-life or reducing the current doses used for IVB may lessen the systemic impact for these premature babies. A prospective, randomized, large-scale study is warranted to elucidate the real impact of IVB on the neurodevelopment of these children.

We observed large changes in the severe developmental delay (MDI/PDI<70) during the study period. There are several possible explanations for these observations. First, the assessments were performed in the patients at the corrected ages of 6, 12, 18, and 24 months. Each period had relevant factors on the assessments that could have caused some variations. Second, the patients of these ages experience fast neurodevelopment, and therefore more neurodevelopmental changes were observed. Third, not all of the patients had the tests performed on them at each time point. These are possible explanations for the large changes in the severe developmental delay in these patients.

This study is limited by the small number of enrolled patients and the non-randomized design. Therefore, we cannot rule out the possibility of selection bias on treatment options, despite the fact that the baseline data among the treatment groups seemed to be similar. In addition, it is possible that that there is another risk factor that predisposes to both more severe ROP and abnormal neurodevelopment but we may not have yet identified. The study is retrospective in its design, and therefore potential counseling bias of the providers is present. Our institution is the largest medical center in Taiwan and one of the major referring centers for severe ROP. We could enroll sicker children and this could contribute to higher incidence of treatment-requiring ROP (Fig 1). The developmental outcomes as assessed by Bayley Scales for Infant Development (II) can only be used as a surrogate of the outcome measure, and may not be completely accurate [33]. The vision data of the patients were not available to correlate with the mental and psychomotor developmental data. However, all of the children had attached retinae with regression of ROP, and their vision was good enough to perform the tasks in the neurodevelopmental survey. The follow-up period of the current study is only 2 years, and during this period some fluctuations in neurodevelopment were noted as the systemic development was still not complete. Therefore, long-term neurodevelopmental data are even more important because they are closer to the final outcome when the development process has matured. Further studies with a randomized design and long-term follow-up and including more patients are needed to further explore the effect of IVB on ROP patients.

## Conclusions

Our results did not show any significant detrimental effects on the neurodevelopment of patients with ROP 2 years after IVB. Detrimental effects were found on the neurodevelopment of the patients who received combined IVB and laser treatment, presumably because they received more procedures and sedation, had earlier PMA for treatment, had more type 1 ROP with more immature retinae, or because destruction of the blood retina barrier after laser treatment caused more IVB leakage into the systemic circulation. However, this was not because these patients had a lower gestational age or birth weight or additional severe systemic comorbidities at baseline. More data are needed to objectively assess the effect of IVB on the neurodevelopment of premature babies.

## Supporting Information

S1 File STROBE statement related to the study. (DOCX)

## References

[1] Alon T, Hemo I, Itin A, Pe'er J, Stone J, Keshet E (1995) Vascular endothelial growth factor acts as a survival factor for newly formed retinal vessels and has implications for retinopathy of prematurity. Nat Med 1: 1024–1028. 7489357 10.1038/nm1095-1024

[2] Early Treatment For Retinopathy Of Prematurity Cooperative Group (2003) Revised indications for the treatment of retinopathy of prematurity: results of the early treatment for retinopathy of prematurity randomized trial. Arch Ophthalmol 121: 1684–1694. 14662586 10.1001/archopht.121.12.1684

[3] Hurwitz H, Fehrenbacher L, Novotny W, Cartwright T, Hainsworth J, Heim W, et al (2004) Bevacizumab plus irinotecan, fluorouracil, and leucovorin for metastatic colorectal cancer. N Engl J Med 350: 2335–2342. 15175435 10.1056/NEJMoa032691

[4] Spaide RF, Laud K, Fine HF, Klancnik JM Jr., Meyerle CB, Yannuzzi LA, et al (2006) Intravitreal bevacizumab treatment of choroidal neovascularization secondary to age-related macular degeneration. Retina 26: 383–390. 16603955 10.1097/01.iae.0000238561.99283.0e

[5] Bashshur ZF, Haddad ZA, Schakal AR, Jaafar RF, Saad A, Noureddin BN (2009) Intravitreal bevacizumab for treatment of neovascular age-related macular degeneration: the second year of a prospective study. Am J Ophthalmol 148: 59–65. 10.1016/j.ajo.2009.02.006 19375689

[6] Spaide RF, Fisher YL (2006) Intravitreal bevacizumab (Avastin) treatment of proliferative diabetic retinopathy complicated by vitreous hemorrhage. Retina 26: 275–278. 16508426 10.1097/00006982-200603000-00004

[7] Mason JO III, Nixon PA, White MF (2006) Intravitreal injection of bevacizumab (Avastin) as adjunctive treatment of proliferative diabetic retinopathy. Am J Ophthalmol 142: 685–688. 17011869 10.1016/j.ajo.2006.04.058

[8] Avery RL, Pearlman J, Pieramici DJ, Rabena MD, Castellarin AA, Nasir MA, et al (2006) Intravitreal bevacizumab (Avastin) in the treatment of proliferative diabetic retinopathy. Ophthalmology 113: 1695–1705. 17011951 10.1016/j.ophtha.2006.05.064

[9] Ruiz-Moreno JM, Montero JA, Lugo F, Amat P, Staicu C (2008) Intravitreal bevacizumab in recurrent diabetic vitreous haemorrhage after vitrectomy. Acta Ophthalmol 86: 231–232. 17888083 10.1111/j.1600-0420.2007.01021.x

[10] Jonas JB, Libondi T, von BS, Vossmerbaeumer U (2008) Intravitreal bevacizumab for vitreous haemorrhage. Acta Ophthalmol 86: 585–586. 18081904 10.1111/j.1600-0420.2007.01107.x

[11] Iliev ME, Domig D, Wolf-Schnurrbursch U, Wolf S, Sarra GM (2006) Intravitreal bevacizumab (Avastin) in the treatment of neovascular glaucoma. Am J Ophthalmol 142: 1054–1056. 17157590 10.1016/j.ajo.2006.06.066

[12] Kriechbaum K, Michels S, Prager F, Georgopoulos M, Funk M, Geitzenauer W, et al (2008) Intravitreal Avastin for macular oedema secondary to retinal vein occlusion: a prospective study. Br J Ophthalmol 92: 518–522. 10.1136/bjo.2007.127282 18211942

[13] Prager F, Michels S, Kriechbaum K, Georgopoulos M, Funk M, Geitzenauer W, et al (2009) Intravitreal bevacizumab (Avastin) for macular oedema secondary to retinal vein occlusion: 12-month results of a prospective clinical trial. Br J Ophthalmol 93: 452–456. 10.1136/bjo.2008.141085 19074916

[14] Jaissle GB, Leitritz M, Gelisken F, Ziemssen F, Bartz-Schmidt KU, Szurman P (2009) One-year results after intravitreal bevacizumab therapy for macular edema secondary to branch retinal vein occlusion. Graefes Arch Clin Exp Ophthalmol 247: 27–33. 10.1007/s00417-008-0916-2 18696094

[15] Sato T, Kusaka S, Shimojo H, Fujikado T (2009) Simultaneous analyses of vitreous levels of 27 cytokines in eyes with retinopathy of prematurity. Ophthalmology 116: 2165–2169. 10.1016/j.ophtha.2009.04.026 19700197

[16] Sato T, Kusaka S, Shimojo H, Fujikado T (2009) Vitreous levels of erythropoietin and vascular endothelial growth factor in eyes with retinopathy of prematurity. Ophthalmology 116: 1599–1603. 10.1016/j.ophtha.2008.12.023 19371954

[17] Mintz-Hittner HA, Kennedy KA, Chuang AZ (2011) Efficacy of intravitreal bevacizumab for stage 3+ retinopathy of prematurity. N Engl J Med 364: 603–615. 10.1056/NEJMoa1007374 21323540 PMC3119530

[18] Micieli JA, Surkont M, Smith AF (2009) A systematic analysis of the off-label use of bevacizumab for severe retinopathy of prematurity. Am J Ophthalmol 148: 536–543. 10.1016/j.ajo.2009.05.031 19660736

[19] Malik S, Vinukonda G, Vose LR, Diamond D, Bhimavarapu BB, Hu F, et al (2013) Neurogenesis continues in the third trimester of pregnancy and is suppressed by premature birth. J Neurosci 33: 411–423. 10.1523/JNEUROSCI.4445-12.2013 23303921 PMC3711635

[20] Breier G, Albrecht U, Sterrer S, Risau W (1992) Expression of vascular endothelial growth factor during embryonic angiogenesis and endothelial cell differentiation. Development 114: 521–532. 1592003 10.1242/dev.114.2.521

[21] Wu WC, Yeh PT, Chen SN, Yang CM, Lai CC, Kuo HK (2011) Effects and complications of bevacizumab use in patients with retinopathy of prematurity: a multicenter study in taiwan. Ophthalmology 118: 176–183. 10.1016/j.ophtha.2010.04.018 20673589

[22] Wu WC, Kuo HK, Yeh PT, Yang CM, Lai CC, Chen SN (2013) An updated study of the use of bevacizumab in the treatment of patients with prethreshold retinopathy of prematurity in taiwan. Am J Ophthalmol 155: 150–158. 10.1016/j.ajo.2012.06.010 22967867

[23] Banach MJ, Ferrone PJ, Trese MT (2000) A comparison of dense versus less dense diode laser photocoagulation patterns for threshold retinopathy of prematurity. Ophthalmology 107: 324–327. 10690834 10.1016/s0161-6420(99)00042-1

[24] Glascoe FP, Byrne KE (1993) The usefulness of the Developmental Profile-II in developmental screening. Clin Pediatr (Phila) 32: 203–208. 7681737 10.1177/000992289303200402

[25] Provost B, Heimerl S, McClain C, Kim NH, Lopez BR, Kodituwakku P (2004) Concurrent validity of the Bayley Scales of Infant Development II Motor Scale and the Peabody Developmental Motor Scales-2 in children with developmental delays. Pediatr Phys Ther 16: 149–156. 17057542 10.1097/01.PEP.0000136005.41585.FE

[26] Jobe AH, Bancalari E (2001) Bronchopulmonary dysplasia. Am J Respir Crit Care Med 163: 1723–1729. 11401896 10.1164/ajrccm.163.7.2011060

[27] Wu WC, Lai CC, Chen KJ, Chen TL, Wang NK, Hwang YS, et al (2010) Long-term tolerability and serum concentration of bevacizumab (avastin) when injected in newborn rabbit eyes. Invest Ophthalmol Vis Sci 51: 3701–3708. 10.1167/iovs.09-4425 20181842

[28] Bakri SJ, Snyder MR, Reid JM, Pulido JS, Singh RJ (2007) Pharmacokinetics of intravitreal bevacizumab (Avastin). Ophthalmology 114: 855–859. 17467524 10.1016/j.ophtha.2007.01.017

[29] Sato T, Wada K, Arahori H, Kuno N, Imoto K, Iwahashi-Shima C, et al (2012) Serum concentrations of bevacizumab (avastin) and vascular endothelial growth factor in infants with retinopathy of prematurity. Am J Ophthalmol 153: 327–333. 10.1016/j.ajo.2011.07.005 21930258

[30] Wu WC, Lien R, Liao PJ, Wang NK, Chen YP, Chao AN, et al (2015) Serum Levels of Vascular Endothelial Growth Factor and Related Factors After Intravitreous Bevacizumab Injection for Retinopathy of Prematurity. JAMA Ophthalmol 133:391–397. 10.1001/jamaophthalmol.2014.5373 25569026

[31] Robinson GS, Ju M, Shih SC, Xu X, McMahon G, Caldwell RB, et al (2001) Nonvascular role for VEGF: VEGFR-1, 2 activity is critical for neural retinal development. FASEB J 15: 1215–1217. 11344092 10.1096/fj.00-0598fje

[32] Martinez-Castellanos MA, Schwartz S, Hernandez-Rojas ML, Kon-Jara VA, Garcia-Aguirre G, Guerrero-Naranjo JL, et al (2013) Long-term effect of antiangiogenic therapy for retinopathy of prematurity up to 5 years of follow-up. Retina 33: 329–338. 10.1097/IAE.0b013e318275394a 23099498

[33] Hack M, Taylor HG, Drotar D, Schluchter M, Cartar L, Wilson-Costello D, et al (2005) Poor predictive validity of the Bayley Scales of Infant Development for cognitive function of extremely low birth weight children at school age. Pediatrics 116: 333–341. 16061586 10.1542/peds.2005-0173

