# Supplementary material for: Neurodevelopmental Outcomes in Infants with Retinopathy of Prematurity and Bevacizumab Treatment
Source: PLoS One. 2016 Jan 27;11(1):e0148019. doi: 10.1371/journal.pone.0148019 (PMC4729687; doi:10.1371/journal.pone.0148019)
Supplement: S1 File — (DOCX) [file pone.0148019.s001.docx]

STROBE Statement—checklist of items that should be included in reports of observational studies

|  | | Item No. | | Recommendation | Page  No. | | Relevant text from manuscript |
| --- | --- | --- | --- | --- | --- | --- | --- |
| **Title and abstract** | | 1 | | (*a*) Indicate the study’s design with a commonly used term in the title or the abstract | 2 | | ***Methods:*** The study design was retrospective observational case series conducted at an institutional referral center. |
|  |  |  |  | (*b*) Provide in the abstract an informative and balanced summary of what was done and what was found | 2 | | Results: Sixty-one patients who finished the neurodevelopmental survey were included. No detrimental effects on neurodevelopment were found in IVB group compared with the patients who received laser treatment only. The patients in the IVB + laser group had a higher incidence of significant mental (p=0.028) and psychomotor (p=0.002) impairment at 24 months than the patients in the laser group. The odds ratio of having severe psychomotor defects in the IVB + laser group was 5.3 compared with the laser group (p=0.041). |
| Introduction | | | | | | |  |
| Background/rationale | | 2 | | Explain the scientific background and rationale for the investigation being reported | 5 | | VEGF has been shown to play an important role in the neurodevelopment of newborn infants,[19,20] and the impact of possible systemic VEGF suppression after IVB on the neurodevelopment of these patients is unknown. |
| Objectives | | 3 | | State specific objectives, including any prespecified hypotheses | 5 | | This study aimed to investigate the neurodevelopment of premature infants with severe ROP up to the age of 2 years after IVB. |
| Methods | | | | | | |  |
| Study design | | 4 | | Present key elements of study design early in the paper | 6 | | This was a retrospective study to assess neurodevelopment in extremely low birth weight (ELBW) infants after IVB or laser treatment for ROP. |
| Setting | | 5 | | Describe the setting, locations, and relevant dates, including periods of recruitment, exposure, follow-up, and data collection | 6, 9 | | The data were collected from Chang Gung Memorial Hospital in Taoyuan, Taiwan,  Patients with type 1 ROP who were treated with laser or IVB treatment from December 2007 to December 2010 were followed and collected for data analysis.  Neurodevelopmental assessments were part of standard care and the data were obtained from medical records. Neurodevelopmental outcomes in the ROP patients after IVB or laser treatment were assessed by Bayley Scales for Infant Development (II).[24]A certified child psychologist who had experienced with this assessment and was blinded to the patient’s prior treatment performed the evaluations for the patients. The assessments were performed in the patients at the corrected age of 6, 12, 18, and 24 months. |
| Participants | | 6 | | (*a*) *Cohort study*—Give the eligibility criteria, and the sources and methods of selection of participants. Describe methods of follow-up  *Case-control study*—Give the eligibility criteria, and the sources and methods of case ascertainment and control selection. Give the rationale for the choice of cases and controls  *Cross-sectional study*—Give the eligibility criteria, and the sources and methods of selection of participants | 6,7,9 | | Patients with type 1 ROP who were treated with laser or IVB treatment from December 2007 to December 2010 were followed and collected for data analysis.  The inclusion criteria for the current study were patients: with type 1 ROP who had received either laser, IVB, or combined treatment, with regression of ROP following treatment; who had been followed up with neurodevelopmental assessments for 2 years; and whose parents had signed consent forms. The patients with major congenital anomalies, and those who lost to follow-up in the 2-year study period were excluded from the study.  A certified child psychologist who had experienced with this assessment and was blinded to the patient’s prior treatment performed the evaluations for the patients. The assessments were performed in the patients at the corrected age of 6, 12, 18, and 24 months. Each period had relevant items on the assessments. |
|  |  |  |  | (*b*) *Cohort study*—For matched studies, give matching criteria and number of exposed and unexposed  *Case-control study*—For matched studies, give matching criteria and the number of controls per case | NA | |  |
| Variables | | 7 | | Clearly define all outcomes, exposures, predictors, potential confounders, and effect modifiers. Give diagnostic criteria, if applicable | 7,8,9 | | Relevant clinical events including Apgar score, hemodynamically significant patent ductus arteriosus that required medical or surgical intervention, necrotizing enterocolitis, bronchopulmonary dysplasia, sepsis, intraventricular hemorrhage (IVH), periventricular leukomalacia (PVL), the rate of inborn, use of antenatal steroids, number of days of ventilator used, respiratory dystress syndrome (RDS), and the postmenstrual age (PMA) of the first treatment were recorded.  A certified child psychologist who had experienced with this assessment and was blinded to the patient’s prior treatment performed the evaluations for the patients. The assessments were performed in the patients at the corrected age of 6, 12, 18, and 24 months. Each period had relevant items on the assessments. The assessments consisted of 2 parts: mental development index (MDI) and psychomotor development index (PDI), and each item was scored by the specialist. |
| Data sources/ measurement | | 8* | | For each variable of interest, give sources of data and details of methods of assessment (measurement). Describe comparability of assessment methods if there is more than one group | 9 | | Neurodevelopmental outcomes in the ROP patients after IVB or laser treatment were assessed by Bayley Scales for Infant Development (II). |
| Bias | | 9 | | Describe any efforts to address potential sources of bias | 9 | | A certified child psychologist who had experienced with this assessment and was *blinded* to the patient’s prior treatment performed the evaluations for the patients. |
| Study size | | 10 | | Explain how the study size was arrived at | NA | | Since this is a retrospective study, study size was not calculated in advance. |
| Continued on next page Quantitative variables | 11 | | Explain how quantitative variables were handled in the analyses. If applicable, describe which groupings were chosen and why | | 10 | Continuous variables were presented as the mean and standard deviation. | |
| Statistical methods | 12 | | (*a*) Describe all statistical methods, including those used to control for confounding | | 10 | Continuous variables were presented as the mean and standard deviation. The chi-square test was applied to examine associations between categorical variables in the study groups. The Shapiro-Wilk test was used to test the normality of the continuous variables in this study. | |
|  |  |  | (*b*) Describe any methods used to examine subgroups and interactions | | 10 | An analysis of variance (ANOVA) was used to compare differences in continuous variables among the study groups, and the Kruskal-Wallis test was used for continuous variables with skew distribution. Fisher's least significant difference test was used for post-hoc pairwise comparisons to identify the groups that were significantly different after ANOVA. | |
|  |  |  | (*c*) Explain how missing data were addressed | | 11 | When comparing cross-sectional neurodevelopmental outcomes among study groups, the missing data were excluded in the analyses. | |
|  |  |  | (*d*) *Cohort study*—If applicable, explain how loss to follow-up was addressed  *Case-control study*—If applicable, explain how matching of cases and controls was addressed  *Cross-sectional study*—If applicable, describe analytical methods taking account of sampling strategy | | 7 | In this study, patients with loss to follow-up were excluded in the analysis. | |
|  |  |  | (*e*) Describe any sensitivity analyses | | 11 | Multiple logistic regression analysis was performed to compare poor neurodevelopmental outcomes among the study groups by adjusting infant sex, gestational age, and birth weight. | |
| Results | | | | | | | |
| Participants | 13* | | (a) Report numbers of individuals at each stage of study—eg numbers potentially eligible, examined for eligibility, confirmed eligible, included in the study, completing follow-up, and analysed | | 11 & Fig 1 | Sixty-one premature, ELBW patients who completed the neurodevelopmental assessments up to 2 years of age were included and entered into the final analysis. | |
|  |  |  | (b) Give reasons for non-participation at each stage | | NA |  | |
|  |  |  | (c) Consider use of a flow diagram | | NA |  | |
| Descriptive data | 14* | | (a) Give characteristics of study participants (eg demographic, clinical, social) and information on exposures and potential confounders | | 12,13 & Table 1 | There were no differences in the gestational age at birth (p=0.098), birth weight (p=0.168), sex (p=0.112), Apgar score at 1 minute after birth (p=0.677), Apgar score at 5 minutes after birth (p=0.778), the rate of hemodynamically significant patent ductus arteriosus (p=0.772), the rate of necrotizing enterocolitis (p=0.411), the rate of sepsis (p=0.945), the rate of grade 1 or 2 IVH (p=0.521), the rate of grade 3 or 4 IVH (p=0.633), PVL (p=0.239), the rate of inborn (0.801), use of antenatal steroids (0.484), number of days of ventilator used (p=0.569) among the 3 study groups. The presence of zone 1 ROP was significantly higher in the IVB + laser group (p=0.034). The rates of having zone 1 ROP were 15.2%, 25%, and 50% for the laser, IVB, and the IVB + laser groups, respectively. Bronchopulmonary dysplasia was present in all of the patients according to the definition of a previous paper,[26] and therefore the data were not entered into the statistical analysis. The demographics of the patients as well as the associated systemic risk factors are listed in Table 1. | |
|  |  |  | (b) Indicate number of participants with missing data for each variable of interest | | Table 2, Figs 2 & 3 |  | |
|  |  |  | (c) *Cohort study*—Summarise follow-up time (eg, average and total amount) | | 11 | Sixty-one premature, ELBW patients who completed the neurodevelopmental assessments up to 2 years of age were included and entered into the final analysis. | |
| Outcome data | 15* | | *Cohort study*—Report numbers of outcome events or summary measures over time | | Table 2 |  | |
|  |  |  | *Case-control study—*Report numbers in each exposure category, or summary measures of exposure | | NA |  | |
|  |  |  | *Cross-sectional study—*Report numbers of outcome events or summary measures | | NA |  | |
| Main results | 16 | | (*a*) Give unadjusted estimates and, if applicable, confounder-adjusted estimates and their precision (eg, 95% confidence interval). Make clear which confounders were adjusted for and why they were included | | 11 & Table 3 | Odds ratio was adjusted for sex, gestational age, and birth weight of the patients. These confounders were adjusted because they are relevant to future neurodevelopmental outcome. | |
|  |  |  | (*b*) Report category boundaries when continuous variables were categorized | | NA |  | |
|  |  |  | (*c*) If relevant, consider translating estimates of relative risk into absolute risk for a meaningful time period | | NA |  | |

Continued on next page

| Other analyses | 17 | Report other analyses done—eg analyses of subgroups and interactions, and sensitivity analyses | 16, 17 | The odds of having severe psychomotor developmental defects (PDI<70) were 4.7 for the IVB group and 5.3 for the IVB + laser group compared with the laser treatment group. The analysis did not reach statistical significance comparing the IVB group with the laser group (p=0.075), however the analysis did reach statistical significance when comparing the laser + IVB group with the laser group (p=0.041) |
| --- | --- | --- | --- | --- |
| Discussion | | | | |
| Key results | 18 | Summarise key results with reference to study objectives | 18 | Our results show that the patients in the IVB + laser group had a higher incidence of severe psychomotor developmental defects than the patients in the laser treatment alone group. The direct comparisons of MDI or PDI in the patients treated with laser treatment alone and IVB alone did not show significant differences in mental or psychomotor development up to 2 years of follow-up. Though there were no statistically significant differences found in the neurodevelopmental scores in the IVB group compared to laser group, there were certainly increased OR of abnormal MDI and PDI (at max effect) in IVB group (p value of 0.084 and 0.075). It is therefore a possible safety concern of using IVB on ROP patients and it is not known whether these effects will become more or less evident as we keep following up these patients. To the best of our knowledge, this paper is the first to evaluate the systemic development of children in children of Chinese decent after IVB with 2 years of longitudinal follow-up. |
| Limitations | 19 | Discuss limitations of the study, taking into account sources of potential bias or imprecision. Discuss both direction and magnitude of any potential bias | 21, 22 | This study is limited by the small number of enrolled patients and the non-randomized design. Therefore, we cannot rule out the possibility of selection bias on treatment options, despite the fact that the baseline data among the treatment groups seemed to be similar. In addition, it is possible that that there is another risk factor that predisposes to both more severe ROP and abnormal neurodevelopment but we may not have yet identified. The study is retrospective in its design, and therefore potential counseling bias of the providers is present. Our institution is the largest medical center in Taiwan and one of the major referring centers for severe ROP. We could enroll sicker children and this could contribute to higher incidence of treatment-requiring ROP (Figure 1). The developmental outcomes as assessed by Bayley Scales for Infant Development (II) can only be used as a surrogate of the outcome measure, and may not be completely accurate [33]. The vision data of the patients were not available to correlate with the mental and psychomotor developmental data. However, all of the children had attached retinae with regression of ROP, and their vision was good enough to perform the tasks in the neurodevelopmental survey. The follow-up period of the current study is only 2 years, and during this period some fluctuations in neurodevelopment were noted as the systemic development was still not complete. Therefore, long-term neurodevelopmental data are even more important because they are closer to the final outcome when the development process has matured. Further studies with a randomized design and long-term follow-up and including more patients are needed to further explore the effect of IVB on ROP patients. |
| Interpretation | 20 | Give a cautious overall interpretation of results considering objectives, limitations, multiplicity of analyses, results from similar studies, and other relevant evidence | 20, 21 | The assessment of neurodevelopment after IVB for ROP has been conducted by few studies. Martínez-Castellanos et al^[32]^ did not show any neurodevelopmental impact 5 years after the use of IVB. Banker et al (Banker AS; Banker DA. Anatomical, functional, OCT and neurodevelomental analysis outcomes of Intravitreal Bevacizumab injection without laser for retinopathy of prematurity: 6 year follow-up. APAO, Hyderabad, India 2013) also did not find any signs of developmental delay in infants 6 years following the use of IVB. However, these studies and ours were limited by a small number of patients and a non-randomized study design. Other anti-VEGF agents with a shorter systemic half-life or reducing the current doses used for IVB may lessen the systemic impact for these premature babies. A prospective, randomized, large-scale study is warranted to elucidate the real impact of IVB on the neurodevelopment of these children. |
| Generalisability | 21 | Discuss the generalisability (external validity) of the study results | 21, 22 | Therefore, long-term neurodevelopmental data are even more important because they are closer to the final outcome when the development process has matured. Further studies with a randomized design and long-term follow-up and including more patients are needed to further explore the effect of IVB on ROP patients. |
| Other information | |  | | |
| Funding | 22 | Give the source of funding and the role of the funders for the present study and, if applicable, for the original study on which the present article is based | 23 | This study was supported by grants from the Ministry of Science and Technology, Taipei, Taiwan (NSC101-2314-B-182A-055-MY3 and 104-2314-B-182A-100-MY2) and Chang Gung Memorial Hospital, Taoyuan, Taiwan (CMRPG3D0251 and CMRPG3E0521). These funding organizations play no role in “design and conduct of the study; collection, management, analysis, and interpretation of the data; and preparation, review, or approval of the manuscript; and decision to submit the manuscript for publication. |

*Give information separately for cases and controls in case-control studies and, if applicable, for exposed and unexposed groups in cohort and cross-sectional studies.

**Note:** An Explanation and Elaboration article discusses each checklist item and gives methodological background and published examples of transparent reporting. The STROBE checklist is best used in conjunction with this article (freely available on the Web sites of PLoS Medicine at http://www.plosmedicine.org/, Annals of Internal Medicine at http://www.annals.org/, and Epidemiology at http://www.epidem.com/). Information on the STROBE Initiative is available at www.strobe-statement.org.
